# Understanding and Evaluating the Implementation of Integrated Care: A ‘Three Pipe’ Problem

**DOI:** 10.5334/ijic.2609

**Published:** 2016-12-31

**Authors:** Nick Goodwin

**Affiliations:** IJIC, 10 Goldsmith Close, Bicester, UK

In their 2004 systematic review on the diffusion of innovations in service organizations, Greenhalgh *et al* concluded that there was a lack of any robust understanding in how complex health service innovations can be implemented and sustained (or not) across contexts and settings [1]. An underlying implication from this work was the need for more ‘realistic evaluation’ methodologies to help unpick how outcomes may result from the intricate interplay between multi-component interventions in different contexts and settings [2].

One such advance has been the recent development of the COMIC Model for the comprehensive evaluation of integrated care interventions [3]. Derived from work undertaken in the recently concluded EU-funded Project INTEGRATE [4], the COMIC Model (Context, Outcomes and Mechanisms of Integrated Care interventions) utilised the realistic synthesis approach to study the interplay between contexts, mechanisms and outcomes across selected case examples of integrated care, including for diabetes in Dutch Care Groups. The authors were able to demonstrate how such an approach brought insights into understanding how, when and why integrated care interventions influenced outcomes in these specific cases.

As a conceptual tool, realistic synthesis provides a useful template to provide an in-depth narrative description of the various factors that may influence outcomes and, potentially, to then take the lessons from one evaluation and test them across a range of different contexts. Indeed, other work within Project INTEGRATE formulated a benchmarking tool by creating a set of generic factors influencing the implementation of integrated care that appears to have face validity across condition-specific groups (e.g. diabetes, COPD, geriatric conditions and mental health) and across different contexts and settings of deployment [4].

In this edition of IJIC, a special collection of perspective papers on the building blocks of integrated care has shed further light on some of the more critical components [5,6,7,8,9,10]. What these amply demonstrate is that the successful implementation of integrated care requires an effective composition of a complex set of interventions at the micro-, meso- and macro-levels. Moreover, effective implementation is as much relational as it is technical. In other words, the influence of pre-existing values, cultures, politics and relationships (both personal and organisational) play as much a significant part in influencing outcomes as the role of technical components such as governance structures, financial incentives, organisational and service models, workforce skills or the ability to engage and empower people in their care.

The main conclusion to be reached is that, whilst we have come a long way in being able to articulate the key building blocks of integrated care, the interplay between them is so complex and intertwined that it seems an impossible challenge to create any simple implementation model. Yet, if integrated care is to advance, we must become better at smoothing over the many obstacles and challenges to implementation that have bedevilled the uptake and roll-out of even the most proven of integrated care interventions.

This is quite the ‘three-pipe problem’ for integrated care since science has yet to make the real breakthrough to address Greenhalgh *et al’s* original challenge in how we might better understand the implementation and sustainability of complex innovations. Recent attempts [e.g. 3,11,12,13,14] have used a blend of realistic synthesis, behavioural theory and mixed-methods to understand implementation effectiveness.

This ability to understand the effectiveness of implementation strategies is particularly important when it comes to planning and justifying investments. As Tsiachristas *et al* [15] argue in this edition of IJIC, designing economic evaluations for integrated care needs to embrace this complexity since existing approaches that focus on single, or reduced numbers, of implementation elements (and typically also screen out the more complex, multi-morbid and frail populations) will not provide the answers to today’s health and care system challenges. Some innovative conclusions are drawn, such as the potential use of cost-consequence analysis accompanied by Multi-Criteria Decision Analysis, but the future clearly requires further funding for methodological research as well as international collaboration since health care decision-makers need evidence on integrated care today.

As Glasby [10] summarises, the future for integrated care requires partners in care to be much more clear about the purpose and outcomes that they are seeking to produce. Too often it can be the case that no real thinking has been made to the logic behind integrated care activities meaning that expectations are built upon false assumptions (or none at all). This may imply that efforts to promote integrated care are self-serving (e.g. to address a policy or management imperative) rather than being the ‘means to an end’ in improving care and outcomes for people.

Glasby [10] also articulates the central point of this editorial that in integrated care it is often the more intangible qualities of complex interventions that make all the difference. Relationships and values matter whether they are at the interface between professionals and patients, within care teams, or between health and care organisations. Hence, in taking forward change towards integrated care, simultaneous innovation is needed in its technical aspects (e.g. service redesign) *and* its relational aspects (e.g. building support for change and the ability to collaborate). We are only just beginning to understand the challenging implications this brings for decision-makers tasked with leading and managing integrated care innovations in practice.
